# Morphological Changes of Bovine Nasal Chondrocytes Induced by Interleukin-1α

**Published:** 2013-05-05

**Authors:** Maryam Yadegari, Mahmoud Orazizadeh, Mahmoud Hashemitabar, Ali Khodadadi

**Affiliations:** 1 Department of Anatomical Sciences, Cellular and Molecular Research Center (CMRC), Faculty of Medicine, Ahvaz Jundishapur University of Medical Sciences, Ahvaz, Iran; 2 Department of Immunology, Faculty of Medicine, Ahvaz Jundishapur University of Medical Sciences, Ahvaz, Iran

**Keywords:** Chondrocytes, Interleukin-1α, Morphology, Extracellular Matrix, Cartilage

## Abstract

**Objective:**

A study of the histological events under interleukin-1α (IL-lα) induction of bovine nasal cartilage (BNC) could result in useful data to better understand the mechanisms involved in tissue breakdown in joint diseases. The aim of this study was to investigate the effects of IL-lα on chondrocyte phenotype and extracellular matrix (ECM) changes in BNC explants.

**Materials and Methods:**

In this experimental study, samples were divided into two groups. Group I (control group) BNC explants were cultured only in Dulbecco’s modified Eagle’s medium (DMEM). In group II, BNC explants were treated with IL-lα (10 ng/ ml) for 28 days. Then, samples were harvested on culture days 3, 7, 14, 21 and 28 and chondrocyte morphology and ECM alterations were assessed by invert microscopy and histology by hematoxylin and eosin (H&E) and Alcian blue. Cell viability was evaluated by the lactate dehydrogenase (LDH) assay test. Data were analyzed by the t test and p<0.05 was considered significant.

**Results:**

IL-lα induced significant morphological changes in cartilage. In the presence of IL-lα, most chondrocytes transformed into a fibroblast-like morphology with a granular black point appearance. An increase in the cell: matrix ratio was observed and there were decreased numbers of chondrocytes.IL-lα induced breakdown of ECM. We observed partial degradation of ECM between days 7-14 and complete degradation occurred between days 21-28 of culture. The LDH levels increased.

**Conclusion:**

IL-1α induced morphological changes in chondrocytes and increased destruction of cartilage ECM. There was a parallel correlation between proteoglycan degradation and changes in chondrocyte morpholgy.

## Introduction

Degenerative joint disorders such as rheumatoid arthritis (RA) and osteoarthritis (OA) are characterized by the destruction of articular cartilage (1, 2). Articular cartilage is composed of extracellular matrix (ECM) and chondrocytes, which are sparsely distributed throughout the matrix and appear to play an important role in the pathogenesis of joint diseases (3, 4). Cartilage ECM is composed of collagen, proteoglycans, non-collagenous proteins, and other macromolecules responsible for matrix organization and maintenance (5, 6). Proteoglycans constitute about 4-10% of the total wet weight and are a mix of large aggregating (50-85%) and large non-aggregating (10-40%) proteoglycans (5, 7-9).

In joints disorders, chondrocytes initiate the release of proteoglycan and collagen from the tissue in response to pro-inflammatory cytokines; degradation of these components are principal features of articular cartilage damage (6, 10, 11). Previous studies have confirmed the presence of interleukin- 1 (IL-l), as a pro-inflammatory cytokine in synovial tissue and fluids of patients with RA and in OA joints (10, 12, 13). Some studies assessed different mechanisms of IL-1on cartilage degradation, such as induction of matrix metalloproteases (MMPs) and the release and inhibition of transforming growth factor-β, and chondrocyte proliferation. IL-1 also increased nitric oxide (NO) activity, which has been shown to induce apoptosis of chondrocytes (6, 8, 10, 11, 14, 15).

Chondrocytes in articular cartilage have a normal round shape. There are multiple interactions between the chondrocyte and ECM. These interactions are critical to the biological functions of chondrocytes, such as the synthesis and degradation of ECM components and differentiation. Chondrocytes are responsible for the synthesis, maintenance and maturation of the matrix within which they are embedded (3, 10). It has been previously shown that in the absence of supporting matrix, chondrocytes lose their ordinary normal morphology and become fibroblast-like dedifferentiated cells. These cells express type I rather than type II collagen and cease synthesis of aggrecan (16-18). The dedifferentiated chondrocytes gradually shift from the synthesis of large aggregating proteoglycans (aggrecan) to low molecular weight proteoglycans (versican) (16-18). The chondrocytes under a variety of mechanisms actively alter their surrounding matrix. These alterations are mediated through receptor molecules and intracellular signaling pathways (19).

Kozaci et al. have shown that IL-1α induced fibroblast-like chondrocyte morphology in a culture of BNC explants (10). Although numerous studies have focused on the degradation effects of interleukin-1α (IL-lα) on cartilage, no general consent has been achieved on the possible mechanisms with which IL-lα affect chondrocyte morphology, proteoglycan degradation and their correlation. Because chondrocytes have an important role in the pathogenesis of joint diseases, thus elucidation of chondrocyte morphological changes could result in useful data and clear mechanisms of tissue degradation in these diseases (3, 4).

In our previous study, IL-lα induced matrix breakdown in bovine nasal cartilage (BNC) explants and created a disease model of RA (20). The most commonly used tissue in cartilage degradation is cartilage derived from the bovine nasal, which is available in large quantities and responds rapidly to various cytokines. This cartilage is a suitable model of cartilage destruction for investigating the mechanisms of cartilage catabolism (6, 8, 10). In this study we have used BNC explants as a source for chondrocytes and ECM components and evaluated their interactions in joint disorders. Then, BNC explants were incubated with IL-1α and its effects on the cartilage component and chondrocyte morphology were determined by invert microscopy and histology techniques.

## Materials and Methods

### Chemicals

In this experimental study we obtained human recombinantinterleukin-1α from Gibco (PHC0017). Dulbecco’s modified Eagle’s medium (DMEM) was also obtained from Gibco, UK. Glutamine, penicillin G, streptomycin, amphotericin B, and L-ascorbic acidwere all obtained from Sigma.

### Preparation of cartilage and explant culture

Bovine nasal septa were obtained from a local abattoir and processed for culture shortly after slaughter. The nasal septum was dissected out and samples thoroughly washed with normal saline and sterile phosphate-buffered saline (PBS) (20). The connective tissue sheath was removed from the cartilage with a sterile scalpel. The tissue was washed four times with 2000 U/ml penicillin G and 0.1 mg/ml streptomycin plus 2.5 µg/ml amphotericin B, and once again with the latter that contained ten times the concentration of penicillin and streptomycin. The samples were punched by a sterile 2 mm diameter punch tool. The uniform slices were cultured in serum-free DMEM that contained 2000 U/ml penicillin G, 0.1 mg/ml streptomycin, 2 mM glutamine, 2.5 µg/ml amphotericin B, and 50 µg/ml L-ascorbic acid for 28 days in 24-well sterile plate at 37 ˚C in a humidified atmosphere of 5% CO_2_ and 95% O_2_.

### Experimental design

The samples were divided into two groups. In group I (control),explants were cultured only in DMEM and group II(experimental) explants were treated with IL-1α (10ng/ml) for 28 days. Lyophilized human IL-1α was reconstituted in sterile deionized H_2_O. IL-lα was added to DMEM before the media was transferred to the culture wells. Plates were incubated at 37˚C. Four wells for each group were considered and each well contained two pieces of cartilage in 400 µl of medium. At days 3, 7, 14, 21, and 28, we harvested the samples after which they were assessed for morphology and changes in ECM by invert microscopy, histology and light microscopy.

### Chondrocyte morphology and ECM breakdown assay

Morphological alterations of unfixed BNC explants in different groups were assessed and visualized by invert microscopy.

### Histology assessment

A number of samples from the different groups were collected and fixed in 10% formaldehyde, after which their morphological changes were assessed histologically and by light microscope. Formaldehyde-fixed and paraffin-embedded 6 µm sections were stained with hematoxylin and eosin (H&E) (10). Digital histographic images were captured using an Olympus BH-2 microscope.

### Proteoglycan degradation in BNC cultures

BNC samples were fixed in 10% formaldehyde, dehydrated through increasing concentrations of ethanol, then embedded in paraffin. Next, 6 µm sections were stained by the Alcian blue staining technique (21). Appropriate fields of view from the samples were evaluated for proteoglycan degradation. Digital histographic images were captured using an Olympus BH-2 microscope.

### Measurement of lactate dehydrogenase activity

As an indicator of cell viability, the amount of cytoplasmic enzyme lactate dehydrogenase (LDH) in the culture medium was measured on different culture days. An optimized LDH test (Roche) was used to quantify LDH activity in the medium of the cartilage explant cultures. The media from the control and experiment groups on days 3, 7, 14, 21 and 28 (100 µl/well) were carefully removed and transferred into corresponding wells of a 96-well flat bottom microplate. Absorbance was measured using an ELISA reader according to the manufacturer’s instructions.

### Statistics

Statistical significance of differences was assessed with the t-test by SPSS for Windows (version 15), followed by the post hoc Tukey comparison test. P<0.05 was considered statistically significant. Data are shown as mean ± SEM for each group

## Results

### Effects of interleukin-1α (IL-lα) on chondrocyte morphology

**Fig 1:**
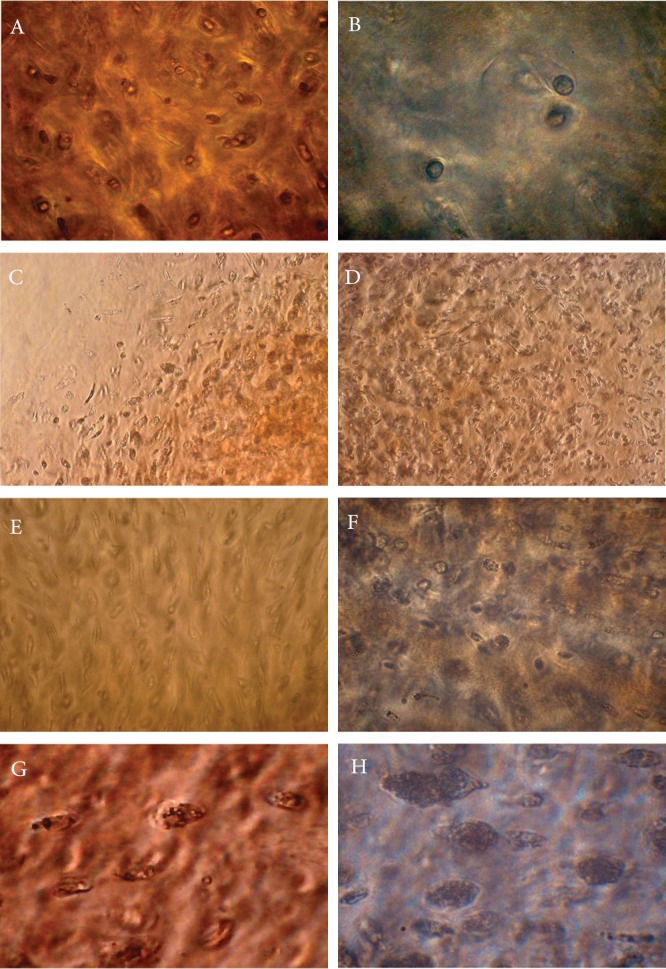
Effects of IL-1α on chondrocyte morphology in explant culture. Explants were assessed by invert microscope. A. Cartilage explant from the control group after 28 days. B. Cartilage explant cultured for 3 days in the presence of IL-1α. C. Cartilage explant cultured for 7 days in the presence of IL-1α. The edges of the cartilage explants started to become translucent. D. Cartilage explant cultured for 14 days in the presence of IL-1α. E and F Cartilage explant cultured for 21 days in the presence of IL-1α. At day 21, the cell membranes began to show a vesicular appearance and the cytoplasm showed a granular appearance . At day 28 of culture, chondrocytes showed a granular black point appearance (G and H). Magnification×400 for images A, B, E-H; ×200 for images C and D.

We assessed the effects of IL-lα on chondrocyte morphology by invert microscope. In the control group that was cultured only with DMEM, chondrocytes had a spherical shape in an intact ECM after 28 days of culture (Fig 1A). In the presence of IL-lα, explants had distinct morphological changes in the ECM and chondrocytes. In the presence of IL-lα, significant morphological changes were observed.

At day 3 there were negligible morphological alterations in the chondrocytes (Fig 1B). After 7 days, we observed heterogeneity in shape and translucence of the cartilage explant edges in the chondrocytes (Fig 1C). After 14 days, an increase in the chondrocyte: matrix ratio was observed due complete disappearance of the edges of the cartilage matrix (Fig 1D). At day 21, most cells showed fibroblast-like morphology with a foamy, vacuolated cytoplasm (Fig1E), a vesicular appearance of the membrane and granular appearance of the cytoplasm (Fig 1F). By day 28 of culture the chondrocytes were floating in the culture medium and we observed complete breakdown of the ECM around the cells. Chondrocytes showed a granular black point appearance (Figs 1G and 1H). The explants in the experiment group gradually changed to loose, translucent pieces from day 14; at day 28 they had completely dissolved.

### Histological assessment

**Fig 2:**
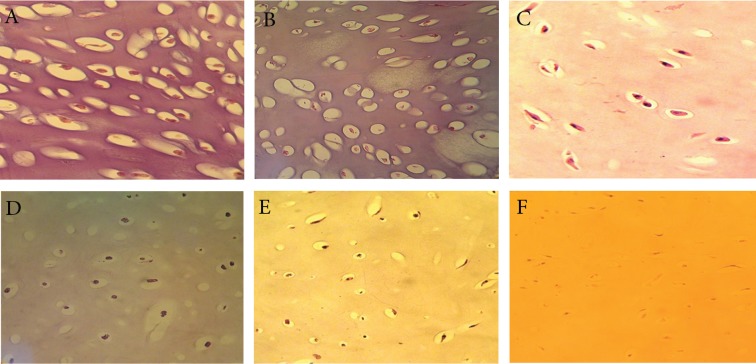
Effects of IL-1α on tissue characteristics in bovine nasal explants. The samples were sectioned and stained with H&E. A. Section from the control group after 28 days. B. Section from a cartilage explants cultured for 3 days in the presence of IL-1α. C. Section from a cartilage explant cultured for 7 days in the presence of IL-1α. D. Section from a cartilage explant cultured for 14 days in the presence of IL-1α. E. Section from a cartilage explant cultured for 21 days in the presence of IL-1α. F. Section from a cartilage explant cultured for 28 days in the presence of IL-1α. Magnification × 400.

Morphological alterations were assessed by H&E staining (Fig 2). In the control group ECM was intact; the chondrocytes’ cytoplasm retained a normal appearance at day 28 of culture. There were few numbers of fibroblast-like chondrocytes with pyknotic nuclei compared to the IL-lα treated group (Fig 2A). In the presence of IL-lα, explants showed morphological changes.

At day 3, there were negligible morphological changes to the chondrocytes (Fig 2B). After 7 days, chondrocytes showed variations in cell shape and some had enlarged nuclei. A few numbers of cells had fibroblast-like morphology (Fig 2C). At day 14, most cells contained pyknotic nuclei (Fig 2D). At day 21, most cells showed fibroblast- like morphology with pyknotic nuclei, the matrix became more translucent compared with day 14 and nucleus dislocation was observed (Fig 2E). At day 28, there was a rapid period of almost complete dissolution of the cartilage; most chondrocytes transformed into an elongated fibroblast-like morphology with pyknotic nuclei (Fig 2F).

### Histological demonstration of proteoglycan degradation in interleukin-1α (IL-1α)-induced bovine nasal cartilage (BNC) explants

**Fig 3:**
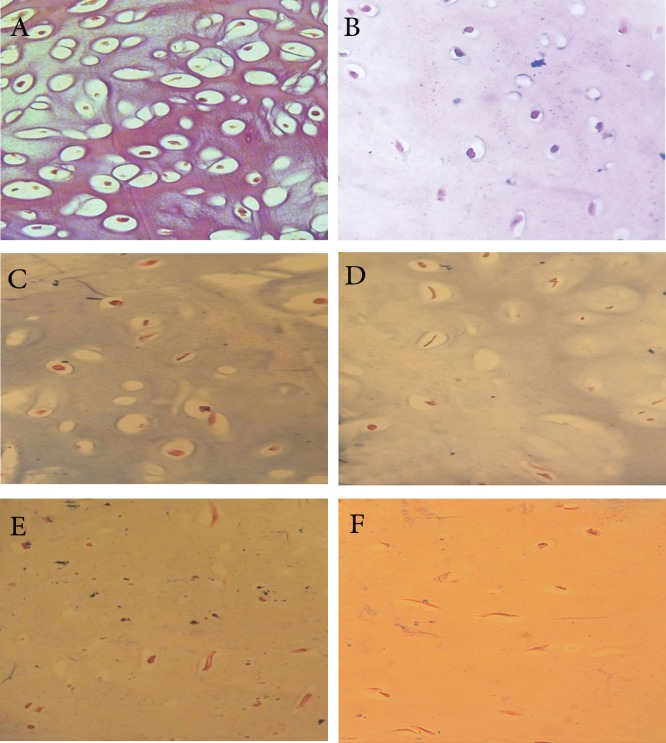
Effects of IL-1α on proteoglycan degradation. The samples were stained with Alcian blue. A. Section from control group after 28 days. B. Section from a cartilage explant cultured for 3 days in the presence of IL-1α. C. Section from a cartilage explant cultured for 7 days in the presence of IL-1α. D. Section from a cartilage explant cultured for 14 days in the presence of IL-1α. E. Section from a cartilage explant cultured for 21 days in the presence of IL-1α. F. Section from a cartilage explant cultured for 28 days in the presence of IL-1α. Magnification ×400.

Proteoglycans in the cartilage were visualized with Alcian blue staining (Fig 3). In the control group, there are relatively intense staining of proteoglycan (Fig 3A). As shown in (Fig B-F), there was a correlation between the incubation day with IL-lα and proteoglycan degradation, and the disappearance of Alcian blue staining. The effect of IL-lα on proteoglycan breakdown was detectable after 7 days of stimulation with IL-l α (Fig 3C). Dark blue staining with Alcian blue disappeared in the presence of IL-lα, which indicated the loss of proteoglycan.

### Viability assay

**Fig 4:**
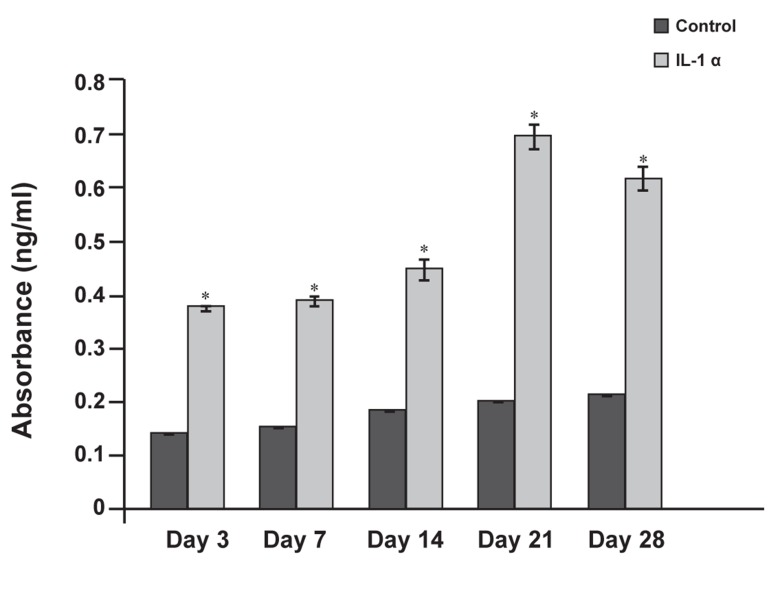
Comparison of lactate levels in media in bovine nasal cartilage (BNC) explants after exposure to IL-1α and in the control group for 28 days. Data represents absorbance. *P<0.001 compared with control group.

Chondrocyte viability in cartilage explants was assessed by screening for the production of LDH using a cytotoxicity detection kit (Roche). There was an increase in lactate levels produced by explants treated with IL-lα compared with the control group (p<0.001; Fig 4).

## Discussion

Joint disorders are associated with increased production of pro-inflammatory cytokines, which are thought to contribute to the pathogenesis of these diseases as well as cartilage degradation. While many reports have suggested that changes to chondrocyte morphology are one of the important steps in joint diseases, the exact mechanism of these alterations is unknown (3, 10).

In the present study, we have investigated the effects of IL-1α on chondrocyte morphology and ECM alterations of BNC by invert microscope and histological assessment at designated time points. IL-lα induced significant degradation of BNC. This model has been frequently used in different studies (20, 22). In the current study, we observed a parallel degradation process in chondrocyte morphology and ECM characteristics.

In the presence of IL-lα, most chondrocytes showed fibroblast-like morphology with a granular black point appearance. Kozaci et al. have shown that IL-1α (50 ng/ml) induced morphological changes in chondrocytes; the maximum effects were seen after two weeks of exposure to IL-lα (10). In support of the Kozaci et al. study, after 7 days of treatment with IL-lα, the chondrocytes in our study began to show phenotypic variations. Other variations were also seen and the edges of cartilage explants started to become translucent. In our study after 14 days there was an increase in the chondrocyte: matrix ratio; the cartilage edges were thoroughly dissolved. At this time during the culture period, it seemed that most chondrocytes were floating in the culture medium. At day 21 of culture there were decreased numbers of cells. Thus, we have concluded that IL-lα inhibits chondrocyte proliferation (23). Another explanation might be because IL-1α induced apoptosis on the chondrocytes (15). In the current study, at day 28, most chondrocytes showed a degenerated pyknotic appearance. Possibly these granular cells lost their membranes. There is a relationship between increased LDH levels and the lack of cell membrane.

The present study results were consistent with those of Kouri et al. who described phenotypic variations in human samples of articular cartilage from the knee of an OA patient according to transmission electron microscopy. These researchers showed that in the OA chondrocytes there were atypical aggregates composed only of secretory cells or degenerated chondrocytes (24). However these morphological changes were not assessed by our time points. In a study by Baici et al. the chondrocytes in severe cases of OA became phenotypically dedifferentiated and appeared to have more fibroblastic figures. Thus, the fibroblastlike morphology of chondrocytes with foamy and vacuolated cytoplasms observed in the current study are in consistent with Baici et al. in severe OA (25).

In the present study, IL-1α also caused chon - drocyte cytoplasm more eosinophilic. According to results of previous studies, chondrocytes in OA cartilage contained more intracellular organelles and indicated the potential for synthesis and secretory activities (25, 10). Consistent with the current study, Shohani et al. have shown that IL-lα (10 ng/ml) significantly induced degradation of cartilage ECM components (20). Their study analyzed cartilage oligomeric matrix protein (COMP) and collagen released in the supernatants.

Proteoglycans and the collagen fibrillar network have profound effects on cartilage ECM and are obviously important to the structural integrity of cartilage(4, 26). Previous studies have shown that IL-1 and tumor necrosis factor alpha (TNF-α) affect the release of proteoglycan from cartilage tissue and block the synthesis of new proteoglycan molecules (4, 6).

In the present study, IL-lα induced proteoglycan degradation. At day 3 proteoglycan degradation was negligible; between days 7 to 14, it increased gradually, and the highest level was observed from days 14 to 21. Kozaci et al. have reported that in cultures of nasal cartilage with IL-lα (50 ng/ml), most of the proteoglycan were released within the first week (6). The present study results were consistent with their results in terms of proteoglycan release, however there was a difference in the timing of proteoglycan release, which could be attributed to the use of different doses of IL-lα in our study. Shingleton et al. have shown that BNC released approximately 30% of glycosaminoglycan (GAG) by day 3 when treated with either retinoic acid (RetA) or IL-1 for 14 days. The effect of combining RetA + IL-1increased the amount of GAG released by day 3 and at day 7 all treatments had induced 80% -90% releasing of the total GAG (11). In addition, these findings were not similar to our results in the timing of proteoglycan degradation because their study used different methods to evaluate changes to ECM. Another possible explanation for this discrepancy might be due to the duration of incubation or the use of different doses of IL-lα in the current study.

According to our results, it seemed that after proteoglycan degradation in the cartilage, morphological changes in the chondrocytes became more prominent. The data suggested that these morphological changes might be related to alterations in the matrix that surrounded the chondrocytes. At day 21, when proteoglycan degradation was apparent and the blue color of matrix disappeared, there was a significant reduction in the number of chondrocytes; all of the cells showed a fibroblast-like morphology. We observed a positive correlation between proteoglycan degradation and changes in chondrocyte morphology. The previous data suggested that loss of proteoglycans might lead to changes in cell morphology (10).

It is possible that IL-lα affects the changes in chondrocyte shape, secondary to the breakdown of ECM. In the presence of IL-1α, chondrocytes in BNC explants appear to be retracted from the edges of their lacunae and start to show nuclear dislocation at day 21 of culture, a time point which was related to significant proteoglycan degradation. Kozaci et al. have suggested that interactions between the ECM and chondrocytes via surface receptors such as integrins could be critical for cellular functions (10, 19). A review of the literature and data has indicated that chondrocytes have actively modified their peri cellular matrix. The modifications include synthesis of new matrix material, modulation of the adhesion of specific receptors, matrix degradation, and direct manipulation of matrix fibrils (10, 27, 28).

## Conclusion

The data of the current study have shown that IL-lα induced significant degeneration in cartilage. It is possible that IL-1α is involved in the mechanisms that lead to degenerative joint disorders. A better understanding of the mechanisms by which IL-1α induces degradation in human chondrocytes can provide a valuable insight into new therapeutic strategies that aim to prevent cartilage destruction. Further studies using electron microscopy or immunohisto chemistry are required to elucidate the proper characteristics of IL-1α and other pro-inflammatory cytokines on chondrocytes. It is necessary to determine the relation between the release of proteoglycans, collagen and the other matrix components into the medium to changes in chondrocyte morphology.
